# Perceived impact of the COVID-19 pandemic on young adults with type 1 diabetes in Rwanda

**DOI:** 10.11604/pamj.2021.40.252.28899

**Published:** 2021-12-21

**Authors:** Jean Claude Habineza, Steven James, Laurien Sibomana, Emma Klatman, Etienne Uwingabire, Jayanthi Maniam, Graham David Ogle

**Affiliations:** 1 Rwanda Diabetes Association, Kigali, Rwanda,; 2 University of Rwanda, College of Medicine and Health Science, Kigali, Rwanda,; 3 University of the Sunshine Coast, School of Nursing, Midwifery and Paramedicine, Petrie, Queensland, Australia,; 4 University of Pittsburgh, Pennsylvania, United States of America,; 5 Life for a Child Program, Diabetes NSW & ACT, Sydney, New South Wales, Australia

**Keywords:** Africa, COVID-19, diabetes, Rwanda, type 1 diabetes, young adults

## Abstract

**Introduction:**

data on the impact of COVID-19 on people with type 1 diabetes (T1D) in less resourced countries are limited. Our study was undertaken in Kigali, Rwanda, and aimed to investigate and describe the problems and challenges experienced by young adults with T1D resulting from the early phase of the pandemic. The study further aimed to understand the mechanisms being used to solve problems and overcome challenges, and perceived support needs.

**Methods:**

this was a cross-sectional study, with anonymous data (n=52) collected through use of questionnaire. Participants were registered, and attending or receiving diabetes-related healthcare through the Rwanda Diabetes Association clinic.

**Results:**

mean+standard deviation age and T1D duration were 24.0±2.1 and 7.4±3.4 years respectively, with sex distribution unequal (male n=22, 42.3%). Of 43 participants, the COVID-19 pandemic did not significantly affect participants´ access to diabetes management supplies and care. Eight (18.6%) participants experienced difficulties accessing blood glucose testing strips, 13 (30.2%) insulin, and three (7.0%) syringes and pen devices. Thirty-two (74.4%) experienced difficulty in attending standard diabetes healthcare reviews at the clinic setting. Some participants experienced hardship, through a decrease in personal or family income (n=42, 80.8%) and challenges in accessing food (n=34, 65.4%), with thirty (57.7%) participants having decreased meal frequency (p<0.001).

**Conclusion:**

our research illustrates the indirect effects of measures undertaken to curb the spread of COVID-19 on young adults with T1D in Rwanda. Study findings may help inform actions to mitigate negative impacts on T1D care in other crises.

## Introduction

With physiological, psychological and social transitions, movement from adolescence into young adulthood heralds significant life change. These changes make adhering to disease self-management challenging for young adults with type 1 diabetes (T1D). Lack of self-management adherence poses substantial risks for early morbidity and mortality [1]. Challenges around T1D self-management have been amplified since coronavirus disease 2019 (COVID-19) was declared a pandemic in 11 March 2020. Within high-income countries, COVID-19 has negatively impacted T1D self-management, with patient access to diabetes management supplies and care decreasing in some settings [2,3]. High mortality rates have also been reported [4,5]. In a study from England, reported odds ratios for in-hospital COVID-19 related death in people with T1D were 3.51 (vs. 2.03 for type 2 diabetes) [5]. Despite this, the impact of COVID-19 on T1D care in low-and-middle income countries (LMICs), has not been widely documented [2,4,6].

With an estimated population of over 12.6 million, and being one of the most densely populated countries in Africa [7], Rwanda is classified by the United Nations as being a least developed country [8]. When Rwanda recorded its first COVID-19 case on 14 March 2020 [9], the government implemented a full national lockdown four days later, and initiated a contact tracing system. With further lock-downs having since been imposed, Rwanda´s rapid government response and coordinated health-care system have helped the country to record only 20,186 overall cases with 280 deaths from COVID-19 (as of 14^th^ March 2021) [10]. A study was undertaken in Kigali, the capital city of Rwanda, to investigate and describe the problems and challenges experienced by young adult patients with T1D resulting from the early phase of the COVID-19 pandemic. The study aimed to understand the mechanisms being used by young people with T1D and their healthcare providers to overcome challenges, and understand their perceived needs for support.

## Methods

**Study design and setting:** this was a quantitative cross-sectional study, carried out in June-September of 2020 and undertaken at the Rwanda Diabetes Association (RDA) clinic.

**Study population:** the study was advertised to young adults with T1D who fulfilled the following criteria: aged 21-29 years, and registered, attending or receiving diabetes-related healthcare through the RDA clinic, which receives support from the Life For a Child (LFAC) program for persons aged <26 y [11]. Around 296 patients with T1D regularly attend the RDA clinic, irrespective of age, with 178 patients aged 21-29 years. Participants were not excluded based upon the length of time attending the association, nor whether they had any comorbidities. There was no financial incentive to participate.

**Data collection:** a questionnaire was developed that included 34 items. Demographic data were collected on aspects including participants´ age and gender, access to diabetes management supplies and medical care, and solutions to challenges experienced. The study was advertised via word of mouth, email and affiliated local online support groups (such as WhatsApp and Facebook), and the questionnaire was primarily self-administered by participants via Kobo Toolbox (Harvard Humanitarian Initiative, Massachusetts, United States of America). Where data collection via Kobo Toolbox was not practical due to inability to access a smartphone, tablet or computer, internet or any literacy issues, participants were able to nominate their method of survey administration - to self-complete a paper-based version, or administer the survey either by phone or verbally; data were transferred directly into Kobo Toolbox by a trained study investigator. Study material were made available in Kinyarwanda (the local language), Swahili and English.

**Data analysis:** anonymous quantitative data were collected from 52 participants (from June-September 2020). Data were expressed as mean±standard deviation (SD), number and percentage. In calculation of T1D duration, the date of diagnosis was assumed to be 30^th^ June on the reported year of diagnosis. Differences between means were examined with t-tests, using variables chosen based upon clinical and/or theoretical reasons. The McNemer test was used to examine the impact of COVID-19 pandemic (before and during) on acute diabetes-related complications. Differences between proportions were examined using Pearson´s chi-squared statistic followed by a pairwise comparison with “N-1” Chi-squared (X^2^) [12]; multiple comparisons were adjusted using Bonferroni. This test was implemented in MedCalc online software [13]. For all tests, p<0.05 was considered as significant. Not all participants answered all questions. Data from incomplete questionnaires were included in analyses where possible and, unless stated otherwise, data are reported as n=52. Data were analysed using Statistical package for the Social Science (SPSS), version 26 (IBM, New York, United States of America) software.

**Ethical considerations:** informed consent was obtained from all participants. Approval to undertake the study was obtained from University of Rwanda, College of Medicine and Health Sciences Human Research Review Ethics Committee, Rwanda (approval reference: 083/CM11S IRB/2020), and the University of the Sunshine Coast, Human Research Ethics Committee, Australia (approval reference: A201405).

## Results

Participant characteristics are provided in Table 1.

**Table 1:** participant characteristics

Variable	n (%) unless stated
Male	22 (42.3)
Age, mean±SD years	24.0±2.1
T1D duration, mean±SD years	7.4±3.4
Place of residence	
Kigali	30 (57.7)
Another city	7 (13.5)
A town/village	15 (28.8)

**Figure 1:**
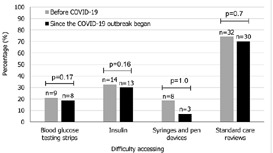
difficulty in access

**Access:** before COVID-19 (March 2020), most (n=44, 84.6%) participants obtained some or all their diabetes management supplies for free or at reduced cost, predominantly (n=36, 81.8%) from the RDA clinic. Few had experienced difficulties in accessing diabetes management supplies and care (Figure 1). The pandemic did not significantly affect participants´ difficulty accessing diabetes management supplies and care (Figure 1). However, of 36 participants, they did report experiencing difficulty in terms of increases in the price of their (undefined) medical supplies (n=16, 44.4%), and that social distancing or lockdown orders impacted the ability to travel (n=13, 36.0%). Twenty-one percent (n=7 of 34) noted a lack of medical supply availability/stock. Participant numbers obtaining diabetes management supplies from the RDA clinic had increased from 69.2 to 90.4% (n=47). After the nationwide lockdown came into force, some participants (n=17, 32.7%) faced problems with law enforcement when trying to access their diabetes supplies and attend diabetes healthcare appointments, in ways they had not prior experienced. Of 47 participants, COVID-19 impacted the way that some participants received their T1D care, with components now utilizing social messaging such as WhatsApp or Facebook (n=1, 2.1%), video conferencing technology such as telephone appointments (n=7, 14.9%), and use of short messaging service (n=4, 8.5%).

**Transport and travel time:** before COVID-19, participants used a variety of transport modes to access their T1D care. Seventy-one per cent (n=37) said the most frequent mode utilised was a car, with 11 (21.2%) undertaking travel that involved bicycle or motor-cycle, and a further 11 (21.2%) travelling by foot. Travel times widely varied, with some (n=19, 36.5%) usually having to travel for 30-60 minutes. Ten participants (19.2%) travelled for 2-3 hours, and a further 10 (19.2%) for >3 hours. After the nationwide lockdown, the number of participants utilising a car decreased (n=31, 59.6%), with 20 (38.5%) reporting now travelling by foot, and 10 (19.2%) as utilising a bicycle or motor-cycle. Travel times remained relatively similar, taking 19 (36.5%) participants 30-60 minutes, 12 (23.1%) 2-3 hours, and seven (13.5%) >3 hours.

**Figure 2:**
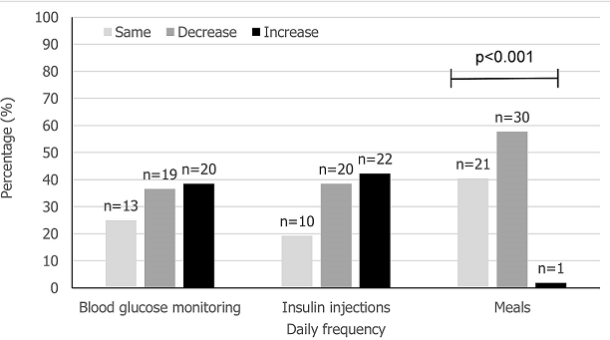
changes to diabetes management practices

**Blood glucose and insulin:** after the nationwide lockdown, the frequency of daily blood glucose monitoring and insulin injections changed in most participants, however there was no tendency towards a reduction in testing or injections (Figure 2).

**Income, food security and lifestyle:** most participants (n=42, 80.8%) reported a decrease in personal or family income since the pandemic began, with 34 (65.4%) reporting that they experienced more challenges in accessing food. Most (n=30, 57.7%) reported a reduction in meal frequency (p<0.001) (Figure 2). Fifty-one participants answered questions about whether their lifestyle changed due to the lockdown. Twenty-two (43.1%) reported a decrease in physical activity, 12 (23.5%) difficulty maintaining social relationships such as with friends, 31 (60.8%) negative perceptions around how healthily they were eating, and 26 (51.0%) an increase in levels of anxiety and stress.

**Acute T1D complications:** of 40 participants, over half said that impacts of the lockdown contributed to experiencing acute complications of T1D, with 21 (52.5%) having experienced non-severe hypoglycaemia. A substantial number experienced severe hypoglycaemia (n=14, 35.0%) or diabetic ketoacidosis (n=4, 10.0%).

**Future outlooks:** participants (n=40, 76.9%) were more (vs. less) concerned about their access to diabetes management supplies and care during the remainder of 2020. Of 40 participants, concerns included maintaining access to insulin (n=26, 65.0%), insulin delivery devices (n=15, 37.5%), blood glucose testing strips (n=17, 42.5%) and standard diabetes healthcare care reviews (n=25, 62.5%).

## Discussion

This study looked at the problems and challenges experienced by young adult patients with T1D during the COVID-19 pandemic. The study aimed to understand the mechanisms being used by young people with T1D and their healthcare providers to overcome challenges, and understand their perceived needs for support. Its findings provide novel information on the problems being experienced by young, vulnerable people with T1D who rely on diabetes assistance programs to access care during the time of a lockdown amidst the COVID-19 pandemic. The lockdown in Rwanda did not affect participants´ access to diabetes management supplies and care for young patients attending the RDA clinic in Kigali. Most participants experienced a decrease in personal or family income, and others faced other hardships. Despite this, some of the young Rwandans with T1D in this study found innovative solutions to counter challenges. They adapted their self-management practices, made use of social messaging services for reviews, and utilized any limited quantities of diabetes supplies and meals in the best way they could. Barriers around access to T1D care in less-resourced settings have been well documented [14-16], and various studies have reviewed access to diabetes supplies, healthy foods, diabetes education and skilled medical care. As COVID-19 has emerged only in the last year, few studies have addressed this challenge to date. Access to healthy foods is critical for glycaemic control in people with T1D [17]. Together with exercise and insulin administration, a proper diet is necessary, incorporating maximum nutrition, with monitoring intake of carbohydrates, protein, and fat. In our study almost two-thirds (65.4%) of participants reported that they experienced more challenges in accessing food after the pandemic began, with over half of the study participants (59.5%) reporting a change in meal frequency. This is concerning considering almost one-fifth of the Rwandan population were already estimated to be food insecure prior to COVID-19 [18]; impacts felt by the pandemic appear to have made this worse.

Rwanda instituted a lockdown early (18 March 2020), and while it was essential from a public health perspective to curb community spread of the virus, services for T1D and access to care were impacted because of the lockdown. The resilience employed by young people had helped them to maintain access to diabetes supplies and care. This situation, in Rwanda, has intensified the need for external assistance, and LFAC has provided funds to the RDA for the provision of diabetes management supplies, healthcare and food. Similar needs have been reported in other countries [2]. Regardless of setting, any failure to implement timely measures to protect individuals with diabetes may ultimately impact upon health and health systems [19]. The use of a diabetes identification card that was implemented by the RDA as part of general diabetes care helped young people to travel internally. While in our study the use of identification to facilitate movement had not been approved with law enforcement leaders as part of a larger initiative, this concept highlights a possible mechanism that could potentially be widely adopted. There are some limitations to our study, namely the use of self-reported data and potential for responder bias. However, these factors are intrinsic to survey design. The increased support provided by LFAC may account for participants reports of decreased difficulty in accessing diabetes management supplies. Finally, the study also involved youth who predominantly live in Kigali, and as such the results might not be country representative.

## Conclusion

Our research illustrates the indirect effects of measures undertaken to curb the spread of COVID-19, particularly the nationwide lockdown, on young people with T1D in Rwanda. Although some participants experienced hardship in reduction and access to, and frequency of healthy meals, many adapted to counter challenges faced around access, characteristic of many people with T1D who do so on a daily basis, even without COVID-19. Study findings may help inform actions to mitigate negative impacts on T1D care in other crises.

### What is known about this topic

- *Lack of type 1 diabetes self-management adherence poses substantial risks for early morbidity and mortality;*
- *Challenges around type 1 diabetes self-management and care have been amplified since the coronavirus disease 2019 was declared a pandemic;*
- *The impact of the coronavirus disease 2019 on type 1 diabetes self-management and care in low-and-middle income countries has not been widely documented*.

### What this study adds

- *Our research illustrates the indirect effects of measures undertaken to curb the spread of COVID-19, on young adults with type 1 diabetes in Rwanda;*
- *Findings may help inform actions to mitigate negative impacts on type 1 diabetes care in other crises*.
